# Democratising clinical trials research to strengthen primary health care

**DOI:** 10.1016/S2214-109X(24)00513-8

**Published:** 2025-03-26

**Authors:** Christopher C Butler, Robert Mash, Nina Gobat, Paul Little, Mpundu Makasa, Martha Makwero, Edward J Mills, Regina Wing-Shan Sit, Max O Bachmann

**Affiliations:** a Nuffield Department of Primary Care Health Sciences, University of Oxford, Oxford, UK; b Division of Family Medicine and Primary Care, Stellenbosch University, Stellenbosch, South Africa; c Community Readiness and Resilience Unit, World Health Organisation, Geneva, Switzerland; d Primary Care Research Centre, University of Southampton, Southampton, UK; e Department of Community and Family Medicine, School of Public Health, University of Zambia, Lusaka, Zambia; f Department of Family Medicine, Kamuzu University of Health Sciences, Blantyre, Malawi; g Department of Health Research Methods, Evidence and Impact, McMaster University, Hamilton, ON, Canada; h The Jockey Club School of Public Health and Primary Care, Faculty of Medicine, The Chinese University of Hong Kong, Hong Kong Special Administrative Region, China; i Norwich Medical School, University of East Anglia, Norwich, UK

## Abstract

The World Health Assembly has called for clinical trials to be strengthened, with broader demographic and geographical inclusion of populations. The objective of this paper is to highlight the importance of rigorous evidence to maximise the health gains of primary health care, and to identify strategies for strengthening clinical trials in primary care. Clinical trials should evaluate interventions of all kinds, including preventive manoeuvres, diagnostics, health service research questions, behavioural and educational interventions, vaccines, therapeutics, and policies. Single question trials can be inefficient and seldom strengthen health systems. New approaches that develop or strengthen health research infrastructure and embed research in primary care will identify effective interventions faster, how to deliver them better, and more accurately determine to whom they should be applied. When patients and community members, together with researchers, contribute to conception, design, and delivery, research will result in more useful, relevant evidence. Traditional site-based recruitment (where the participant comes to the trial) can be complemented by approaches that give people the opportunity to contribute regardless of where they live and receive their health care (taking the trials to the people). However, this cannot be done until regulation is modernised to make it easier for health-care professionals, researchers, and research participants to co-design, deliver, and implement such trials, and to develop processes to coordinate and monitor progress against goals for budget shifts, delivery, engagement, trials activity, and impact. Strengthening primary care trials is especially important in those regions where primary care is most under-resourced and is key to pandemic preparedness. Not doing so risks widening inequities further.

This is the fourth in a **Series** of six papers on clinical trials in global health

## Introduction

WHO states that “primary health care is the most inclusive, equitable, cost-effective and efficient approach to enhance people's physical and mental health, as well as social well-being”.^1^ In the Declaration of Astana, heads of states and governments, and their representatives, committed themselves to “research and share knowledge and experience, build capacity and improve the delivery of health services and care”.^2^ The objective of this paper is to highlight the importance of rigorous evidence to maximise the health gains of primary health care, and to identify strategies for strengthening clinical trials in primary care. We recommend new ways of thinking about trial design and delivery, and coordination of primary health-care research with ongoing monitoring of funding, capacity building, trial activity, engagement, and impact to ensure sustainability.

Primary health care is a systems-wide approach, incorporating primary care delivery (a model of care that supports first-contact, accessible, continuous, comprehensive, and coordinated person-centred care),^1^ empowerment of communities, and multisectoral policy and action on the social and environmental determinants of health.^3^ Primary health care has the potential to improve population health and equity, and save costs through health promotion and disease prevention.^3^ An estimated 75% of the projected health gains from attaining the Sustainable Development Goals could be achieved through primary health care.^1^ Good quality primary health care is a cornerstone and key determinant of population health in any country, and enables social development.^1^, ^4^

In high-income countries such as the UK, over 95% of all contacts between people and health-care services are in primary care, and around 80% of problems are managed at this level.^1^, ^5^ However, universally, access to health care is unequally distributed, disfavouring those in resource-poor circumstances.^6^ Primary care in low-income and middle-income countries (LMICs) typically has fewer resources, poorer infrastructure, hospital-centred health systems, and a greater reliance on fewer professionally trained health-care workers compared to high-income countries.^7^ LMICs often innovate to overcome resource challenges, such as using other cadres of health-care workers. Primary care services are often delivered through informal or semi-formal networks of community health workers and civil society organisations. The COVID-19 pandemic brought a stark reminder of the crucial roles of community health workers and civil society organisations in delivering a wide range of primary care services, including home-based care, sample collection, and vaccine roll-out.^8^, ^9^, ^10^ WHO's revised framework for strengthening the global architecture for Health Emergency Preparedness, Response, and Resilience^11^ calls attention to the need for building resilience of primary care, including through capacity development of the primary health-care workforce to deliver community protection outcomes.

**Key messages**

• Well-developed primary health care is associated with reduced costs, better health outcomes, and equity. For maximum effect, primary health care should be underpinned by evaluations of various interventions in the populations they are intended for, and the World Health Assembly has called for more and better clinical trials that include more diverse populations within and across countries to ensure wider applicability and rigour.
• Traditional clinical trials that are designed to answer a single question can be inefficient and might not strengthen health systems. Trial designs that include master protocols and adaptive features to identify the best approach from a range of options maximise applicability and continuity. Pragmatic trials can give answers about what would happen if interventions were taken up into routine care. Standing clinical research network trials infrastructure that strengthens community capacity for delivery and involvement in trials is crucial to evaluating questions about interventions for non-communicable as well as communicable diseases, including epidemic and pandemic infections. Ways of taking trials research to the people can complement site-based recruitment and enhance research equity and inclusivity.
• New ways of thinking are needed for the identification and prioritisation of research questions, allocation of funding, public engagement, and researcher and site capacity development and regulation, together with a living process of monitoring funding, numbers of registered and completed primary health-care trials, and evidence of uptake of findings into routine care.

The inverse care law states that “the availability of good medical care tends to vary inversely with the need for it in the population served”.^12^ This premise continues to apply within and between countries globally. For example, the African continent has 25% of the global disease burden, but only 3% of the world's professionally trained health workers and less than 1% of the world's health expenditure.^13^ The inverse care law has resonances when it comes to opportunities to participate in research, and this needs to be addressed through strengthening clinical trials to underpin improved primary health care using fresh thinking on trial prioritisation, funding, regulation, clinical trial design, and implementation. This endeavour will involve new ways of funding, designing, and implementing trials, proportionate and appropriately adapted regulation, and embedding trials with ongoing investment in primary health care. Furthermore, permanent and sustainable clinical research network trials research infrastructure, and a trained health workforce, are important to enable epidemic and pandemic preparedness, so that interventions can be evaluated within weeks of an outbreak. Many of the lessons from community-based pandemic trials are applicable to primary care trials research more generally (panel 1).

Panel 1 **PRINCIPLE and PANORAMIC trials of therapeutics in the community for COVID-19**

Rapid identification of effective treatments for use in the community during a pandemic is crucial for the wellbeing of individuals and the sustainability of health-care systems and society. Furthermore, identifying treatments that do not work reduces research wastage, spares people unnecessary side-effects, rationalises the cost of purchasing and stockpiling medication, and reduces inappropriate use of medication. Nevertheless, only a small minority of therapeutic trials for SARS-CoV-2 infections have been in primary care: most opened too late, struggled to recruit, and few produced actionable results. Participation in research is often limited by where a person lives or receives health care, and trial participants might not represent those for whom the treatments are intended. The Platform Randomised Trial Of Treatments In The Community For Epidemic And Pandemic Illnesses (PRINCIPLE) and the Platform Adaptive Trial Of Novel Antivirals For Early Treatment of COVID-19 In the Community (PANORAMIC) trials randomly assigned over 40 000 people with COVID-19 by innovating both trial design (by using novel adaptive platform designs) and trial delivery (by complementing traditional site-based recruitment [the patient comes to the research]). These innovative methods enabled rapid enrolment, eligibility checks, consent, randomisation, microbiological and blood testing, and follow-up without participants having to leave home (taking research to the people), and addressed the so-called inverse research participation law, which highlights disproportionate barriers faced by those who have the most to contribute, and benefit from, research. These trials also transformed the evidence base by evaluating nine medicines to support guidelines and care decisions worldwide for COVID-19 and contribute to antimicrobial stewardship. The PRINCIPLE and PANORAMIC trials represent models of innovation and inclusivity and exemplify the potential of primary care to lead the way in addressing pressing global health challenges.^14^

**Key enablers of this process included:**

• Experience of conducting a platform trial with adaptive capability using Bayesian approaches of a therapeutic for influenza-like illness during peacetime^15^
• Rapid, peer-reviewed funding processes that allowed experienced teams to initiate trials promptly
• Prioritisation of resources from The UK Urgent Public Health Panel for national platform trials to receive priority support for primary care recruitment from The National Institute of Health Research Clinical Research Network infrastructure in England, Health and Care Research Wales, NHS Research Scotland, and the Health and Social Care Board in Northern Ireland
• Using hub and spoke models of recruitment. Over 4500 general practices (spokes) could refer potentially eligible patients to 65 research practice sites (hubs) that would take consent and complete study procedures
• The COVID-19 Therapeutics Advisory Panel independently assessed potential interventions to guide selection
• Early engagement with regulatory bodies, such as the Health Research Authority and the Medicines and Healthcare products Regulatory Authority, facilitated swift trial opening and amendment approvals
• Bringing the study to the attention of potential participants when positive SARS-CoV-2 tests were fed back; regulatory approval for remote eligibility checks using electronic summary care records for some approved medications; remote consent; direct medication delivery to participants’ homes; self-taken swabs and blood spot testing posted to a central laboratory; digitally enabled follow-up with ascertainment of primary outcome complemented with information for participants’ electronic medical records

## The importance of generating evidence for primary health care

Evidence-based interventions delivered in primary health care can have considerable reach and benefits. Effective medication, vaccinations, counselling, policy, social, and behavioural interventions are all essential to primary health care. In addition to person-centred clinical interventions, we also need effective interventions to improve service delivery (eg, community engagement, access, coordination, and person-centredness), health-system inputs (eg, workforce and design of the primary health-care team, health information systems, supply chains) and health-system structures (eg, financing of primary health care, multisectoral action).^16^, ^17^ Such interventions have multiple elements that reflect the complexity of these issues. Evidence is needed not only on the efficacy and effectiveness of interventions, but also on the practicalities of implementation and scale-up.

Despite this, most clinical trials worldwide are conducted in hospital or specialist settings. However, so-called hand-me-down trials evidence from hospital care is often not fit for purpose in primary health care. To ensure maximum applicability, interventions need to be tested in the intended populations and health-care settings where the interventions will be applied, as evidence generated in hospital settings might not be applicable to the diverse populations, disease spectrums, and settings of primary health care. Morbidity in primary health care is different to hospital care: health conditions might be less severe, at an earlier stage of disease progression, less differentiated, and with a greater emphasis on stable chronic care; patients are also more diverse in terms of age, reasons for encounter, and burden of disease. Primary care is also implemented in very different ways: in people's homes, primary care facilities, and emergency rooms, and by health providers with varying capacities and characteristics—from well remunerated, professionally trained, and well supervised health workers, to those that are unpaid, poorly trained, and working with no supervision.^10^ The range of available and suitable medications, imaging, and other diagnostic tests is also different from hospital settings. Patients’ understanding of their conditions and treatments and their access to, trust of, and willingness to engage with health-care professionals also differ considerably, which influences adherence, health behaviour, and outcomes.

## Barriers to performing clinical trials in primary care

Given the importance of clinical trials in contributing to the evidence base for primary health care and the relative lack of such trials, what are the barriers and enablers to implementing clinical trials in primary health care?

Globally, primary care is under considerable resource pressures. Primary care professionals often have less opportunity, capacity, and incentive to participate in research. Contract research organisations (CROs) conduct many of the clinical trials that lead to regulatory approval of a drug, yet rely on existing and often publicly funded networks of trial recruiting sites, predominantly in well-resourced settings. Trial networks associated with CROs and larger academic centres often have well-trained staff capable of the fine-grained requirements of regulatory agencies. In LMICs there are few established primary care trial networks. The scarcity of human resources in LMICs means that most clinical researchers need to balance service delivery with education and research activities, and few have the luxury of prioritising research. In addition, primary care providers are often non-professionally registered (eg, community health workers) or from disciplines with less of a research culture (eg, clinical officers and nurses) and might not have the formal education and registration required for regulatory standard trials. Pharmaceutical companies and CROs have less incentive to conduct non-pharmaceutical trials. They often focus on potentially high-value pharmacological treatments for ongoing treatment of long-term conditions rather than on evaluating delivery strategies in primary health care, for example, and this can entrench inequity by disease.^18^

In contrast, health services research is usually publicly funded, with primary health-care research typically driven by values-based motivation to improve care, rather than by primarily financial-based incentives. This discrepancy means that primary care research is often not prioritised due to time pressures and competing demands. In LMICs, researchers also have limited success with obtaining international grants, and local government funding of primary care research is usually insufficient to support adequately powered, rigorous clinical trials.

The costs associated with clinical trials vary greatly according to whether the intended user is a regulatory agency or a clinical user. Electronic data capture systems required for regulatory approvals are an expense that few primary health-care settings can afford, and so more often they will use academic or free electronic data capture systems that can provide high quality data. Thus, electronic data capture systems using routinely collected clinical data can enhance ascertainment of outcomes such as medically attended consultations and hospital admissions, as well as resource use and longer-term safety outcomes. Data obtained in this way should be accepted and regulations made more proportionate to study risk in this regard.^19^

Research infrastructure is less well developed and accessible in the community. For example, there are 9000 general practices in England, and none of them have research ready pharmacies associated with them.^14^ Rapid access to imaging and specimen sampling is also less developed. In LMICs, primary care clinical research infrastructure is often also weak, with inadequate buildings, lack of space, limited laboratory support, poor telecommunications, and outdated equipment.^20^

Many community-based clinicians and sites, although supportive of research, can be put off by burdensome documentation and required training (often of tangential relevance to being a local principal investigator) and lengthy and complex contracting that sponsors and regulators require even for low-risk studies. These often disproportionate and frequently pointless processes are particularly burdensome for the practices that serve populations living with higher deprivation; these practices are often not as well funded but have high workloads because of the higher burden of ill health that comes with deprivation. Investing in research capacity for a single, short-term study might not be worthwhile for sites, whereas establishing a long-term pipeline of studies and associated funding to address relevant questions will be more attractive.

These factors led us to extend the inverse care law to research opportunities^21^ and to suggest that access to research is often inversely proportional to a participant's potential contribution, and to where and to whom the research findings are most applicable.^14^

## Strategies to enable clinical trials in primary care

Given the various barriers to performing clinical trials, what are the known or potential strategies for overcoming these barriers and enabling such research? Such strategies could be related to policy, finances, planning, education, restructuring, and quality, as well as coordination and monitoring of activity and progress.^22^

National research policy should include a focus on research in primary health care and be based on relevant needs assessments. It should prioritise the key questions on effectiveness that need to be addressed and ensure that adequate finances are available to support clinical trials. The effectiveness of health-system and health services interventions might be as important as the effectiveness of clinical, therapeutics-based interventions focused on specific diseases (panel 2). Doing large-scale, nationwide, and international trials is difficult, with separate institutions and countries sometimes wanting to do their own separate trials for political gain (as happened during the COVID-19 pandemic), and with national funders, regulators, and ethics committees not using harmonised processes, hampering recruitment and uptake of any completed clinical trial.^24^

Panel 2 **Innovation in HIV care delivery**

The Streamlining Tasks and Roles to Expand Treatment and Care for HIV trial addressed the urgent problem in South Africa of high mortality and morbidity among people with HIV whose initiation of antiretroviral treatment (ART) was delayed by shortages of doctors authorised to prescribe it. The trial evaluated a complex intervention comprising nurse initiation and monitoring of ART (NIMART), supported by a clinical decision guide, staff training, organisational strengthening, and managerial and clinical support. The trial was conducted in all 31 primary health-care clinics that provided ART in one province, 16 of which were randomised to receive the intervention. Use of electronic medical records to identify eligible participants and measure outcomes enabled rapid recruitment of over 15 000 participants and completion of the trial in 2 years. With research ethics committee approval, participants’ consent was not sought because it was not feasible and because it would not affect the care individuals received. The trial showed that the intervention did not affect mortality or virological suppression but was associated with better health and HIV programme outcomes. These improvements were despite unanticipated real-world problems including some intervention clinics being unable to deliver NIMART, and a temporary moratorium on ART initiation because of financial restrictions. The trial results coincided with and supported the change of national policy on NIMART, leading to rapid expansion of ART to millions of people with HIV in South Africa, and task shifting in HIV care in other low-income and middle-income countries. Policy relevance and effect was strengthened by long-standing engagement between the researchers and provincial and national health departments, with senior government officials represented on the trial steering committee. This primary care trial exemplifies pragmatic design, efficient use of medical records, appropriate ethical regulation, and timely production of results to influence policy.^23^

National and international coordination are needed for funding, approvals, infrastructure, national prioritisation of questions, health and care resources, access to study sites and patients, and patient intervention recipients.^25^ A major reason why the UK led the world in timely, country-wide trials research during the COVID-19 pandemic was that national platform trials were prioritised by an Urgent Public Health Panel for access to National Health Service recruitment. This endeavour was supported by its standing clinical research network infrastructure in England and analogous standing capability in the other UK devolved administrations. Although this meant that some investigators did not get access to publicly funded facilities for recruitment, it did mean that the UK was able to rapidly set up and recruit enough participants into national trials to rapidly answer important questions. These trials were able to identify which treatments were effective in saving lives and reducing illness burden. They were also able to identify treatments that did not bring benefit but were nevertheless being widely used at the time or carried risk of serious adverse effects, which in turn informed care guidelines and rapidly evolving research directions.^24^, ^26^, ^27^ Advisory panels made recommendations to trialists in the EU and the UK about which treatment to include, ensuring scrutiny with the desired expertise. This approach could usefully be applied to non-pandemic questions, with standing infrastructure seen as a public good, ready to evaluate key prioritised interventions.

Appropriate regulations need to allow for remote eligibility checks whenever appropriate, remote consent, and use of participants’ health data when they have provided a priori consent for this. Currently data protection rules can undermine participant autonomy by denying trial teams access to patient health data for eligibility checks and outcome ascertainment.

Research capacity building remains important, including developing established primary health-care researchers who can champion and lead the field.^28^ The expectation of conducting clinical trials should be included in such capacity building, especially in LMICs. A review of family practice research in Africa found that only 2% of published articles were experimental studies.^29^ A combination of training through educational programmes, short courses for specific skills, and mentoring and collaborations can help build expertise. It is important to recognise that the day-to-day activities of clinical trials require meticulous attention to detail in terms of record keeping. These skills are typically not learned in university settings but require continuing education and accreditation for staff such as database managers, clinical research associates, and site monitors.

For primary care trials to have the greatest benefit, we need to build broader trial leadership and implementation capacity. This endeavour includes supporting and building on ongoing engagement between research staff and government health officials who are responsible for delivering primary care. This engagement is crucial to ensure that the research matches the priorities and challenges of health service providers, that access to primary care facilities and patients is allowed, and that research evidence is translated into large-scale implementation. Engaging local primary care and community-based health workforces in clinical trial implementation brings important benefits for capacity development and income support. These workers are often crucial for the engagement of local communities, and the recruitment and retention of those at higher risk of ill health and poorer outcomes and of groups who are often under-represented in research.^30^ Their role as a trusted intermediary between formal health structures and local communities means they are particularly well placed to raising awareness of trials and to communicate clearly the complexities of trial procedures in contextually and culturally sensitised ways. They can anticipate, identify, and address rumours and misinformation, and strengthen health and research literacy among local populations. Done well, this strengthens trust in science and scientists.^31^ Restructuring the research landscape might be needed with more institutional resources for creating clinical trial or research units that focus on primary health care. Restructuring could also require the development of posts for primary health-care researchers, supportive infrastructure, and equipment.

Analysis of 20 case studies identified four common factors that have had a positive effect on research in primary health care: development of academic departments and appointment of professors of family medicine; direct resources and funding; collaboration between institutions, disciplines, and settings; and the breadth and diversity of research conducted to reflect the scope of primary health care.^32^ Regulation needs to modernise and move away from universal use of regulations intended for high-risk, efficacy, regulatory trials, in highly selected and closely monitored populations, that are often inappropriate and obstructive for lower risk primary care trials. Requiring consent forms to run into several pages that require multiple signatures and patient information leaflets that run into more than 50 pages might provide legal protection for regulators and sponsors but tend to confuse and often frighten potential participants rather than inform them. Rapid initiation of trials in the intended use population is crucial for efficient and timely evidence to guide care and should be facilitated by appropriate regulations. This reform should allow pragmatic trials to incorporate some flexibility in study implementation, given the various ways that care is delivered within and between primary health-care settings.

Although the US Food and Drug Administration and the European Medicines Agency have mandates for their populations, their influence extends well beyond these mandates. With the newly established African Medicines Agency, we hope that they can create guidance of direct relevance to Africa and interact with other LMICs to create relevant guidance of their own.

## Different ways of thinking about trial design in primary care

### Single question studies

Most trials in primary care are small scale, answer single questions, and are thus inefficient. They can take a long time to obtain results and for findings to be implemented. People volunteer, sites are set up, they deliver results, then are often forgotten—the results do not become embedded in care, resulting in wasted infrastructure. Specific study training is required for each new study that does not optimally build experience in trial teams and sites or build community trust in research.

Statistical analysis of this type of trial only begins once a pre-set, fixed sample size is reached, which is usually estimated from trials done in another time and place.^33^ This approach means that recruitment into trials that turn out to have larger than anticipated effect sizes might continue beyond the earliest point at which useful findings could have emerged, had an interim analysis been conducted before the final sample size was reached. Similarly, an equivocal result could be converted to a clear finding, should recruitment have continued beyond the estimated, fixed sample size.^34^ Traditional trials, therefore, do not take data emerging from the trial into account to revise power estimates after randomisation begins. Rather than focusing on which treatment, if any, performs best for a particular condition, such trials only ask one main question: is this intervention more effective than current standard of care or placebo?^35^ Master protocols, including platform clinical trials, on the other hand, focus more on finding the most effective treatment from a number of candidates for a particular illness.

### Adaptive platform trials

An adaptive clinical trial allows for aspects of the trial and statistical procedures to be changed after the trial starts, without undermining validity or integrity.^35^ A platform trial is an adaptive clinical trial in which multiple treatments for the same disease can be tested at the same time, allows for additional treatments to be added while the trial is in progress, and allows for futile interventions to be dropped via frequent interim analyses that ensure each drug remains in the trial only until prespecified thresholds for futility, success, or safety concerns are met.^34^ The trial requires a master protocol that describes the overall study design, with appendices that provide each intervention's details, including additional inclusion criteria and specific monitoring requirements.^36^, ^37^ Specific considerations for engagement and informed consent apply.^31^

### Pragmatic trials

Effectiveness trials should include participants similar to those who might be eligible to receive the treatment should it be found to be effective,^38^ and the process of the study should reflect real-world care as closely as possible. Pragmatic trial design has been advocated for in evaluating the effectiveness of treatments already shown to be efficacious in earlier phase studies that were conducted with more selected patient populations, where internal validity is often high, but applicability can be questionable.^39^, ^40^ Pragmatic trials allow for flexibility in the way an intervention is delivered or care is provided, for example in allowing primary care professionals some discretion in how care is provided to individual patients. Such adapatability contrasts with conventional efficacy trials in which every detail of patient care is predefined. Policy makers and primary care clinicians raise questions regarding the generalisability of efficacy trials to real-world patients, who often have multimorbidity and differ by social determinants of health.^41^

Providing primary care often involves making decisions about the best treatment course in the absence of clear supporting evidence. We might not know, for example, which licensed treatment is best suited to particular patient groups. In the absence of relevant comparative effectiveness studies, clinicians are forced to regularly make arbitrary prescribing decisions, outcome data is not systematically collected, and resultant opportunities for improving care are lost. Thus, randomly assigning patients and conducting follow-up, facilitated by electronic medical records, should be considered best practice in such circumstances, and in some cases should not require individual consent procedures.^42^ This concept is widely agreed upon, but rarely implemented.

Trials that randomise units of care delivery or clusters of people rather than randomising people to a particular treatment are useful for testing systems-wide approaches using routinely collected outcomes, and lead to both efficiencies and challenges of interpretation (panel 2).^43^

### Open trial designs and patient reported outcomes

Placebo control is sometimes assumed to be necessary in all studies to ensure rigour.^44^ However, trials with a comparison group of current practice alone are needed— this is the best way to assess behaviours of patients and clinicians in conditions as close as possible to everyday care,^45^ so that we can answer if the intervention being trialled adds to what already exists. We do not use placebos in primary care, so comparing the care people would receive without a drug is preferable to the artificial situation of care with a placebo. Although open trials do not allow for the assessment of contribution of possible placebo and so-called nocebo effects,^46^ they are likely to provide a better indication of effect sizes and associated costs in routine care. Whilst moderate placebo effects can be anticipated for some conditions, for example chronic pain, there is good evidence that open trials do not provide substantially different estimates from placebo-controlled trials for acute respiratory conditions.^15^, ^47^, ^48^

Despite editorials welcoming pragmatic research,^39^ hospital-based and pharmaceutical industry-based reviewers, as well as journal editors, are more familiar with regulatory studies and tend to be critical of pragmatic, policy-relevant, large-scale trials that include diverse populations. Similarly, some regulators and journal editors shun open trials with patient reported health outcomes in favour of placebo-controlled trials with ostensibly objective outcomes such as examination findings or biomarker levels. However, our goal is often to improve function and wellbeing, and the best way of assessing this is through patient report.

### Practice-based research networks

The Australian Clinical Trials Alliance defines a clinical trial network (CTN) as “a group of clinicians, health professionals, researchers, and often consumers who collaborate to conduct clinical trials in a defined disease or discipline. CTNs conduct and publish multisite clinical trials, to strengthen and improve the evidence base for high-quality health care and/or increase the uptake of evidence into practice.”^49^ The Australian Clinical Trials core principles for CTNs include: collaboration and collegiality including sharing credit for success (eg, group authorship and mutual ownership of outcomes and achievements); alignment of the best interests of researchers, patients, and the CTN; equity; creation of a reusable, sustainable, infrastructure that improves trial quality and feasibility; commitment to improving patient outcomes through generating and implementing evidence derived from trials; conduct of high quality clinical trials that are patient-centred and innovative; and enhancing efficiency of research through coordination of potentially competing trials and prioritisation of research questions.^49^

Practice-based research networks are becoming an important vehicle for primary care research in LMICs.^20^ There are over 40 CTNs in Australia.^49^ In the USA, the Agency for Healthcare Research and Quality has 174 registered CTNs, containing 153 736 clinicians and serving 86 million patients.^50^ Based on trust, CTNs can be a vehicle for conducting studies at scale in real-world settings and enable clinicians to participate in research meaningfully while not being overwhelmed or overcommitted.^51^ The UK Urgent Public Health Panel prioritised resources for national platform trials to receive priority support for primary care recruitment from the National Institute of Health Research (NIHR) clinical research network infrastructure in England, Health and Care Research Wales, NHS Research Scotland, and the Health and Social Care Board in Northern Ireland. This prioritisation of clinicial research network resources was a crucial step towards the focused and successful implementation of the PRINCIPLE^36^ and PANORAMIC^52^ trials of therapeutics for COVID-19 in the community (panel 1). However, without funding and ongoing support of research infrastructure in primary health care, sustainability of CTNs is fragile.

### Taking trials research to the people

The World Health Assembly passed Resolution 75.8 on strengthening clinical trials in 2022, calling for bigger and better trials that include a more diverse range of people, to ensure stakeholder involvement in intervention trials.^38^ Limiting trial participation to those who can attend clinics and hospitals diminishes the applicability of the findings. Estimates of the effectiveness of interventions are often most needed for those who are unable to attend clinic and hospital visits. For example, people with acute infectious diseases who could be very unwell, but who are not admitted to hospital, are usually excluded from trials research that rely on potential participants attending the research site. Those who cannot afford health care similarly could be excluded, thus widening inequalities and reinforcing the further marginalisation of groups who are less often included in clinicial trials. Regulators often require that assessments be done, and samples taken only by health-care professionals, but results from self-sampling with swabs and blood specimens can also provide reliable outcomes.^53^ Routinely collected health-care data can efficiently ascertain particular outcomes. Eligibility can be assessed and consent taken remotely.^54^ However, regulation and licensing bodies might not accept evidence generated in this way, even though these designs are likely to reflect effects more accurately in the real world compared to purely site-based studies.

### Stakeholder and patient and public involvement in trial design

The key to successful trial delivery is a trusting relationship between potential participants, the clinical service, the funders, the trialists, and other key stakeholder groups across the clinical trial ecosystem.^55^ Trust is likely to be developed by embedding research within health-care systems and through an appreciation that engagement to consider participation should be encouraged, particularly among those who have the most to gain from the research findings. Engagement with a wide range of community stakeholders including patient groups, religious organisations, civil society organisations, and community-based organisations is a prerequisite to research that is meaningful and acceptable to those it is intended to benefit and in achieving representative study populations.^56^ Study questions should be formulated together with people that represent the diversity of key populations intended to benefit from trial interventions, and the research methods, materials, and implementation processes should be co-developed with community stakeholders to optimise the acceptability and feasibility of trial procedures.

Explicit standards for public involvement in research have been developed together with tools to facilitate their use. For example, the NIHR in the UK has identified five standards, together with a set of questions for each, to help people and organisations reflect on their progress towards each standard and areas for improvement (panel 3).^57^

Panel 3 **UK standards for public involvement—better public involvement for better health and social care research57**

**Inclusive opportunities**

Offer public involvement opportunities that are accessible and that reach people and groups according to research needs.

• Are people affected by and interested in the research involved from the earliest stages?
• Have barriers to involvement, such as payment for time or accessible locations for meetings, been identified and addressed?
• How is information about opportunities shared, and does it appeal to different communities?
• Are there fair and transparent processes for involving the public in research, and do they reflect equality and diversity duties?
• Is there choice and flexibility in opportunities offered to the public?

**Working together**

Work together in a way that values all contributions, and that builds and sustains mutually respectful and productive relationships.

• Has the purpose of public involvement been jointly defined and recorded?
• Have the practical requirements and arrangements for working together been addressed?
• Have all the potential different ways of working together been explored, and have these plans and activities been developed together?
• Is there a shared understanding of roles, responsibilities, and expectations of public involvement?
• Have individuals’ influence, ideas, and contributions been recognised and addressed?

**Support and learning**

Offer and promote support and learning opportunities that build confidence and skills for public involvement in research.

• Is there a range of support to address identified needs?
• Have specific resources been designated to support learning and development opportunities for the public, researchers, and staff?
• Do the public know where to go for information and support about public involvement?
• Is there a culture of learning by doing, and building on and sharing that learning for researchers, staff, and the public?

**Communications**

Use plain language for well-timed and relevant communications, as part of involvement plans and activities.

• Has a communications plan been developed for involvement activities?
• Are the needs of different people being met through inclusive and flexible communication methods?
• Are processes in place to offer, gather, act on, and share feedback with the public?
• Are you sharing your public involvement learning and achievements, good and bad?

**Impact**

Seek improvement by identifying and sharing the difference that public involvement makes to research. Involve the public in research management, regulation, leadership, and decision making.

• Are the public involved in deciding what the assessment of impact should focus on, and the approach to take?
• Is it clear what information to collect to help assess impact, including who has been involved and how?
• Are there processes in place to help reflect on public involvement?
• Are the changes, benefits, and learning resulting from public involvement acted on?

The James Lind Alliance promotes priority setting partnerships between individuals and organisations representing patients, carers, and health-care professionals to identify research priorities for consideration by funders.^58^ There are five key stages. The first stage is initiation, which involves defining the health or social care issue and agreeing what is in scope, identifying partners, awareness raising, contracting for partnership effectiveness, and establishing a steering group. Second is consultation, where research questions are gathered directly from clinicians, patients, and carers via groups, organisations, or alliances using standard methods such as surveys and focus groups. The third stage, collation, categorises the questions into themes, which are refined and, where required, reworded. Existing evidence bases are checked to establish that prioritised questions are not already answered by up-to-date research reviews. Fourth, prioritisation reduces the research questions to a manageable number and those that are most important. Finally, priority setting workshops are devised and all partner representatives are invited, and a nominal group technique is used.

This approach has been extended for identifying not only priority research questions, but also care needs.^59^ Several scoping studies have already been published to identify research and evidence gaps in primary health care in LMICs.^60^ Progress in funding commitments and developing research teams and infrastructure to fill these gaps is being monitored, but patients and the public were not included in this process. However, even at a global level, patient groups are becoming more organised and could participate.^61^

### Metrics, criteria, and monitoring

Given the potential for health gains from primary health care underpinned by trials research, there is a need to shift research focus to the community. The 30 by 2030 campaign, launched in 2020, called for international donors to assign at least 30% of their vertical, top-down, disease-oriented budgets to strengthening integrated horizontal, community-based systems by 2030, to improve primary health-care services through coordination, increased human resources, and improved infrastructure.^18^

There are good examples of international collaboration between funders on chronic diseases, such as the Global Alliance on Chronic Diseases, which has led to successful task shifting, training, and more comprehensive care for people with non-communicable diseases—but with a disregard for interventions in primary health care that are not specific to non-communicable diseases.^62^ Could we have a global alliance for primary health-care strengthening, with a focus on embedded integrated primary health-care research? An alliance of this kind could lead advocacy, oversee ongoing identification of research gaps, and monitor progress.

A primary health-care strengthening initiative could be developed together with patients and the public to ensure a sustained upward trajectory in commitment and spend from funders both nationally and globally for primary health-care research. Such an initiative could allow for monitoring of the numbers of applications for funding, community engagement in identifying and prioritising questions and effective delivery, researcher and site capacity development, numbers of registered and completed trials, and evidence of uptake of findings into routine care.^63^ Key aspects include: long-term partnerships and working within local systems and constraints; tailoring interventions to local context and needs; service development and operational research that is prioritised, designed, conducted, and replicated when it is embedded within ministry of health and national programmes; designing interventions from the outset for scale-up through careful piloting; responsiveness to windows of funding for scale-up; pragmatic evidence generation; and the use of tested and easily usable tools, training materials, and processes for use in scale-up.^64^

Frameworks for evaluating fidelity of trials of complex interventions usually focus on the interventions described in the protocol. Complex Interventions Design Delivery Receipt (CONSIDER) is a new framework that covers design (the core elements of the intervention), delivery (the actual implementation of the protocol and insertion integrity including the actions of the intervention providers), and receipt (the exposure of the trial participants to the intervention and their response to it). CONSIDER also covers evaluating what was intended to be delivered, what was actually delivered given the complexities of context and variation in primary health-care providers, care pathways and resources, and how study participants responded to the exposure, which allows for intervention refinement with more efficient uptake into routine care.^65^

## Conclusion

Designing and implementing high-quality primary health care is a global priority for universal health coverage and the Sustainable Development Goals. Stronger primary health-care research is needed to address the effectiveness of clinical care, health services, and health systems. Clinical trials are an essential part of the research landscape, yet primary health care often relies on evidence conducted in hospitals and other well-resourced settings. Barriers to clinical trials in primary health care include the scarcity of available expertise, disproportionate and inappropriate regulation, limited data collection technology, poor availability and biased distribution of research funding within and between countries (especially in LMICs), and inadequate standing infrastructure. Strategies to enable clinical trials and overcome these barriers involve policy and regulatory reform, ongoing bottom-up planning of research priorities, realignment of funding, restructuring of research architecture to build research capacity in primary health care, and monitoring of the nature, quality, and effect of clinical trials. These strategies could be facilitated by an international leadership and coordination body that gains commitment from funders and health services to set targets and follow through sustainably.

Primary care trials research can be further strengthened by shifting funding models from a single study question for each trial to trials infrastructure that is embedded in health and care services. Adaptive and platform trials, that have the capacity to evaluate multiple treatments in parallel and in series, are particularly suited for evaluating therapeutic and health service interventions for a wide range of conditions in primary care including communicable and non-communicable illness. By combining traditional community-based sites in primary care with taking research directly into people's homes, we can address the inverse research participation law. Primary health-care trials therefore need to be prioritised, coordinated, mandated, resourced, sustained, simplified, embedded in primary care, and, thus, democratised.

### Contributors

## Declaration of interests

CCB was Chief Investigator of the PRINCIPLE trial. CCB and PL were co-Chief Investigators of the PANORAMIC trial. MOB was co-Chief Investigator of the STRETCH trial. All other authors declare no competing interests.
