# Correction to *Lancet Glob Health* 2025; 13: e749–58

**DOI:** 10.1016/S2214-109X(25)00239-6

**Published:** 2025-06-26

*Butler CC, Mash R, Gobat N, et al. Democratising clinical trials research to strengthen primary health care.* Lancet Glob Health *2025; **13:** e749–58*. In the Acknowledgments section of this Series paper, additional statements pertaining to the funding received for the development of this manuscript and others in the Series have been added. These corrections have been made as of June 26, 2025.

